# PARP1 Inhibition Augments UVB-Mediated Mitochondrial Changes—Implications for UV-Induced DNA Repair and Photocarcinogenesis

**DOI:** 10.3390/cancers12010005

**Published:** 2019-12-18

**Authors:** Csaba Hegedűs, Gábor Boros, Eszter Fidrus, Gréta Nikoletta Kis, Miklós Antal, Tamás Juhász, Eszter Anna Janka, Laura Jankó, György Paragh, Gabriella Emri, Péter Bai, Éva Remenyik

**Affiliations:** 1 Department of Dermatology, Faculty of Medicine, University of Debrecen, 4032 Debrecen, Hungary; hegeduscsaba88@gmail.com (C.H.); fepont92@gmail.com (E.F.); janka.eszter.a@gmail.com (E.A.J.); gemri@med.unideb.hu (G.E.); 2 BioNTech RNA pharmaceuticals GmbH, BioNTech AG, 55131 Mainz, Germany; borosgabor27@gmail.com; 3 Department of Anatomy, Histology and Embryology, Faculty of Medicine, University of Debrecen, 4032 Debrecen, Hungary; greta@anat.med.unideb.hu (G.N.K.); antal@anat.med.unideb.hu (M.A.); juhaszt@anat.med.unideb.hu (T.J.); 4 Department of Medical Chemistry, Faculty of Medicine, University of Debrecen, 4032 Debrecen, Hungary; janko.laura90@gmail.com (L.J.); baip@med.unideb.hu (P.B.); 5 MTA-DE Lendület Laboratory of Cellular Metabolism Research Group, University of Debrecen, H-4032 Debrecen, Hungary; 6 Department of Dermatology and Department of Cell Stress Biology, Roswell Park Comprehensive Cancer Center, 665 Elm St, Buffalo, NY 14203 USA; 7 Research Center for Molecular Medicine, Faculty of Medicine, University of Debrecen, 4032 Debrecen, Hungary

**Keywords:** UVB, PARP, mitochondria, metabolism, biogenesis, autophagy, carcinogenesis, DNA repair

## Abstract

Keratinocytes provide the first line of defense of the human body against carcinogenic ultraviolet (UV) radiation. Acute and chronic UVB-mediated cellular responses were widely studied. However, little is known about the role of mitochondrial regulation in UVB-induced DNA damage. Here, we show that poly (ADP-ribose) polymerase 1 (PARP1) and ataxia-telangiectasia-mutated (ATM) kinase, two tumor suppressors, are important regulators in mitochondrial alterations induced by UVB. Our study demonstrates that PARP inhibition by ABT-888 upon UVB treatment exacerbated cyclobutane pyrimidine dimers (CPD) accumulation, cell cycle block and cell death and reduced cell proliferation in premalignant skin keratinocytes. Furthermore, in human keratinocytes UVB enhanced oxidative phosphorylation (OXPHOS) and autophagy which were further induced upon PARP inhibition. Immunoblot analysis showed that these cellular responses to PARP inhibition upon UVB irradiation strongly alter the phosphorylation level of ATM, adenosine monophosphate-activated kinase (AMPK), p53, protein kinase B (AKT), and mammalian target of rapamycin (mTOR) proteins. Furthermore, chemical inhibition of ATM led to significant reduction in AMPK, p53, AKT, and mTOR activation suggesting the central role of ATM in the UVB-mediated mitochondrial changes. Our results suggest a possible link between UVB-induced DNA damage and metabolic adaptations of mitochondria and reveal the OXPHOS-regulating role of autophagy which is dependent on key metabolic and DNA damage regulators downstream of PARP1 and ATM.

## 1. Introduction

Mitochondria regulate their shape, number, distribution, mass, content of mitochondrial DNA (mtDNA), and metabolic capacity in a process called mitochondrial biogenesis, which requires the orchestration of complex transcriptional control of both nuclear and mitochondrial genes [1,2]. The function of mitochondrial biogenesis is to provide quality control of mitochondria by regulating mitochondrial fission, fusion, and mitophagy [3,4] to maximize the energy utilization of mitochondria [5] to meet cellular and environmental demands. Imbalances or perturbations in these processes can lead to mitochondrial dysfunction [3,6].

Accumulating evidence suggests that mitochondria also play a central role in skin physiology. Although, involvement of other organ systems predominates in classical mitochondrial disorders, several cutaneous diseases can be linked to mitochondrial dysfunctions [7]. Interestingly, mitochondria lack functional nucleotide excision repair (NER) pathway [8,9] which is responsible for the removal of ultraviolet (UV)-induced DNA lesions including cyclobutane pyrimidine dimers (CPD). Accumulation of these DNA photoproducts in mtDNA leads to mutations and deletions resulting in mitochondrial alterations which have been associated with photoaging [10,11] and are present in melanoma [12], as well as in non-melanoma skin cancers [13,14]. The other types of mitochondrial alterations such as upregulated oxidative phosphorylation (OXPHOS), mitochondrial membrane hyperpolarization, and decreased mitophagy are frequently observed in patients with DNA repair deficiencies [15,16,17,18]. Even though, these DNA repair proteins are encoded by the nuclear genome, their absence lead to mitochondrial functional changes emphasizing the importance of nucleus-to-mitochondria (NM) signaling [19].

Growing evidence suggests that the key component of NM signaling is poly (ADP-ribose) polymerase 1 (PARP1) activation [19,20]. PARP1 is a multifunctional zinc-finger protein involved in the regulation of DNA repair, chromatin structure, cell cycle, calcium homeostasis, transcription regulation, cell death, immune response, and metabolism [21,22]. Through either direct interaction or via poly (ADP-ribose) (PAR) polymer formation PARP1 can modulate the activity of ataxia-telangiectasia mutated kinase (ATM) [23] and tumor suppressor protein 53 (p53) [24,25] involved in DNA damage response. Enhanced PARP1 activity also induces ATP depletion [26], which implies the activation of AMPK-activated protein kinase (AMPK) [27,28] that is responsible for the regulation of various cellular pathways including protein kinase B (AKT) and mammalian target of rapamycin (mTOR) [29,30,31]. This complex interplay between PARP1, ATM, AMKP, p53, AKT, and mTOR indicates that these DNA damage responders can fine-tune and modulate the interaction between DNA repair pathways with metabolism [32]. PARP1 and PARP2 also modulate mitochondrial activity through NAD^+^ depletion [33,34,35]. Pharmacological inhibition or deletion of PARP1 and PARP2 improves mitochondrial function and protects against mitochondrial [36,37], metabolic [34], and neurological diseases [38]. PARP inhibitors became valuable tools in treating cancer cells harboring DNA repair defect with the combination of either radio- or chemotherapy [39,40,41]. In addition to several orally-administered PARP inhibitors that are under active clinical development, recently ABT-888 (veliparib) has emerged as an effective drug in treating various solid tumors [42] partially via modulating mitochondrial activity [43,44].

Although UV radiation has been shown to trigger morphological [45,46] and functional changes of mitochondria [47,48,49], published data led to contradictory results most likely due to the diversity of applied UV spectrum, UV dose, and cell type. Studies using UVC irradiation revealed that UVC induced mitochondrial hyperfusion and resulted in enhanced ATP synthesis via oxidative phosphorylation (OXPHOS) [50]. Other authors demonstrated that UVC irradiation caused a significant increase in mitochondrial content, oxygen consumption, and fatty acid oxidation [28]. Nevertheless, the functional consequences of mitochondrial alterations after UVB-induced DNA damage and the molecular pathways leading to mitochondrial changes remain to be elucidated.

In this study, we defined the central role of PARP1 and the linked molecular pathways in mediating UVB-induced DNA damage response and mitochondrial changes in a clinically relevant human keratinocyte cell line.

## 2. Results

### 2.1. PARP Inhibition Impairs CPD Repair, Augments UVB-Induced Cell Cycle Block, Apoptosis and Reduces Keratinocyte Proliferation

**Figure 1:**
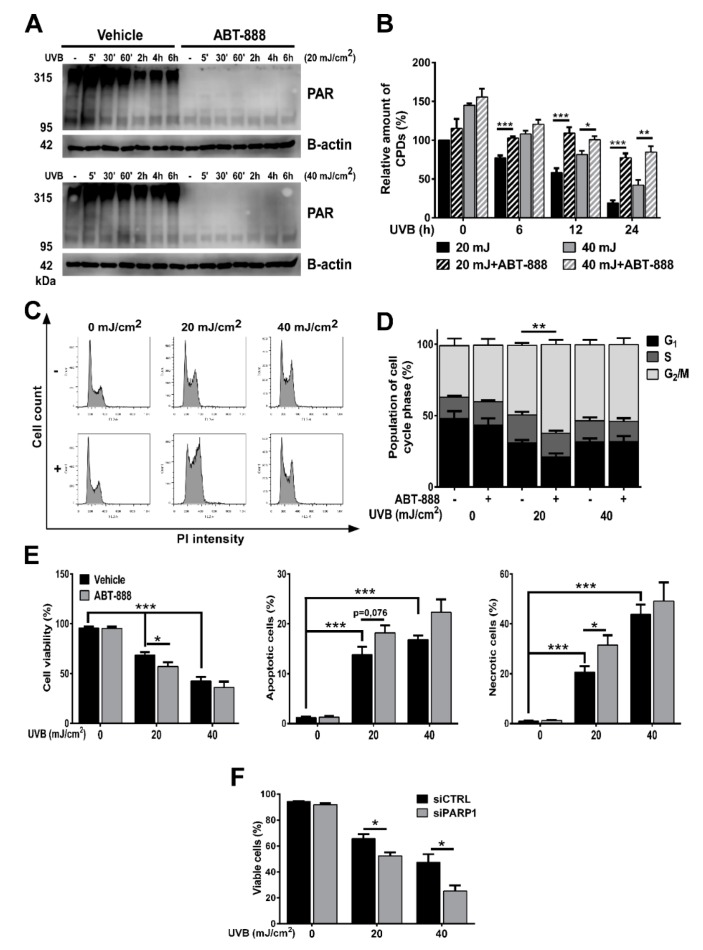
Poly (ADP-ribose) polymerase (PARP) inhibition impairs cyclobutane pyrimidine dimer (CPD) repair, augments ultraviolet B (UVB)-induced cell cycle block and apoptosis. (**A**) Time-course of PARP activity (PAR) after 20 and 40 mJ/cm^2^ UVB exposure and 25 µM ABT-888 were analyzed by Western blot (*n* = 3). (**B**) Cells were exposed to a single dose of 20 or 40 mJ/cm^2^ UVB and collected at various time points for DNA extraction. CPD formation was determined by ELISA (*n* = 4). (**C**,**D**) Cell cycle progression was evaluated by propidium iodide staining after 24 h. DNA content was analyzed in FL2-A (*n* = 4). (**E**) Cell viability, apoptosis, and necrosis was assessed by dual labelling with Annexin V-Alexa 488 and propidium iodide 24 h post-UVB. Double negative cells are considered as viable (*n* = 5). (**F**) Cell viability was measured similarly as in Figure 1E after PARP1 knockdown (*n* = 3). −/+ represent vehicle (−) or ABT-888 (+) treatment *; ** and *** indicate statistically significant difference at *p* < 0.05 and *p* < 0.01, *p* < 0.001, respectively. Error bars represent SEM.

**Figure 2:**
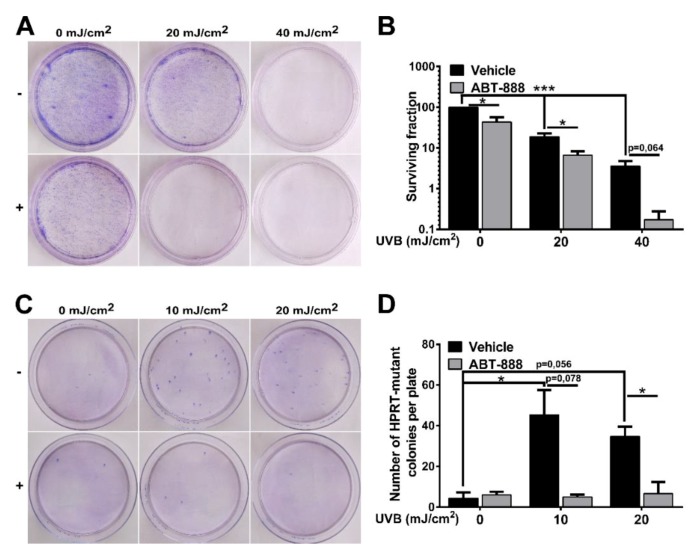
Poly (ADP-ribose) polymerase (PARP) inhibition decreases clone formation and ultraviolet B (UVB)-induced mutation rate. (**A**,**B**) Colony formation assay of HaCaT cells after 10 days post-UVB exposure was assessed by clonogenic assay (*n* = 4). (**C**,**D**) HPRT mutation assay was carried out on CHO cells. Preselected hypoxanthine-guanine phosphoribosyltransferase (HPRT) mutant cells were cultured for 10 days post-UVB (*n* = 3). −/+ represent vehicle (−) or ABT-888 (+) treatment * and *** indicate statistically significant difference at *p* < 0.05 and *p* < 0.001, respectively. Error bars represent SEM.

To explore the effect of UVB on PARP activation, firstly, we investigated poly (ADP-ribose) polymer (PAR) formation in a time-dependent manner in human immortalized keratinocytes exposed to mid (20 mJ/cm^2^) or high (40 mJ/cm^2^) dose of UVB (Figure 1A). In HaCaT cells, poly (ADP-ribosyl) ation (PARylation) signal was initially observed at and over 95 kDa at 5 min after UVB exposure and the extent of PARP activation was dose-dependent with higher PARP activation after 40 mJ/cm^2^ UVB exposure. The signal was lost when cells were treated with ABT-888, a pan-PARP inhibitor (for uncut PAR western see Supplementary Figure S2). PARP1 is considered to contribute 85–90% to total PARP activity, the rest is largely the activity of PARP2 [51,52,53]. Moreover, the size of the PARylated band suggests PARP1 (auto) PARylation indicating the involvement of PARP1 in UVB-induced damage. Since PARP1 is involved in regulation of various DNA repair pathways, we wanted to assess how PARP inhibition (PARPi) regulates the removal of cyclobutane pyrimidine dimers (CPDs) introduced by UVB (Figure 1B). A slow decline of CPDs after UVB exposure was observed, reflective of nucleotide excision repair (NER) activity. In contrast, the relative amount of CPDs in PARP-inhibited cells remained elevated even after 24 h compared to the UVB-irradiated cells suggesting impaired efficiency of NER, similarly as described by King et al. [54]. Since unrepaired photolesions can initiate cell cycle block to prevent cells with DNA damage from entering mitosis, we performed cell cycle analysis (Figure 1C,D). A higher proportion of PARP-inhibited cells after 20 mJ/cm^2^ UVB accumulated in G_2_/M phase of cell cycle characteristic of enhanced DNA damage [21]. ABT-888 treatment sensitized cells to apoptosis after UVB as reflected by decreased cell viability 24 h post-UVB compared to controls (Figure 1E). Gene silencing of PARP1 showed similar changes regarding cell viability as PARP inhibition. Furthermore, long-term keratinocyte survival using May-Grünwald-Giemsa staining in clonogenic assay showed that ABT-888 treatment led to a marked decrease in the number of keratinocyte clones after UVB-irradiation (Figure 2A,B). To find out whether PARPi-induced retention of CPDs induce higher mutation rate, we performed hypoxanthine-guanine phosphoribosyltransferase (HPRT) mutation assay (Figure 2C,D). We could not adjust the method for HaCaT keratinocytes as these cells were extremely tolerant to the 6-thioguanine selection medium. Therefore, we used Chinese hamster ovary (CHO) cells which is a widely used cell line for HPRT mutation assay [55,56,57] and also show high level of p53 protein level due to p53 mutation [58,59] as HaCaT cells do [60]. In this case, we used lower UVB doses to allow the accumulation of mutations compared to higher UVB which potentially triggers apoptosis. Although, both 10 and 20 mJ UVB resulted in increased number of HPRT-mutated cells, interestingly PARP inhibition caused a marked reduction in the number of mutated cell colonies suggesting that PARPi initiate apoptosis of cells with high CPD content instead of allowing the accumulation of gene mutations. Interestingly, some DNA damage markers, including cell viability, CPD and colony formation, cell cycle progression showed no significant difference between the vehicle and ABT-888 treated groups after 40 mJ/cm^2^. This phenomenon can be due to the fact that 40 mJ/cm^2^ UVB dose in our experiments represents such high DNA damage that cannot be augmented by PARP inhibition. However, PARP1 knockdown cells displayed significantly lower cell viability compared to control siRNA-transfected cells even after 40 mJ/cm^2^ UVB irradiation.

### 2.2. PARP Inhibition Enhances UVB-Mediated Mitochondrial Biogenesis

**Figure 3:**
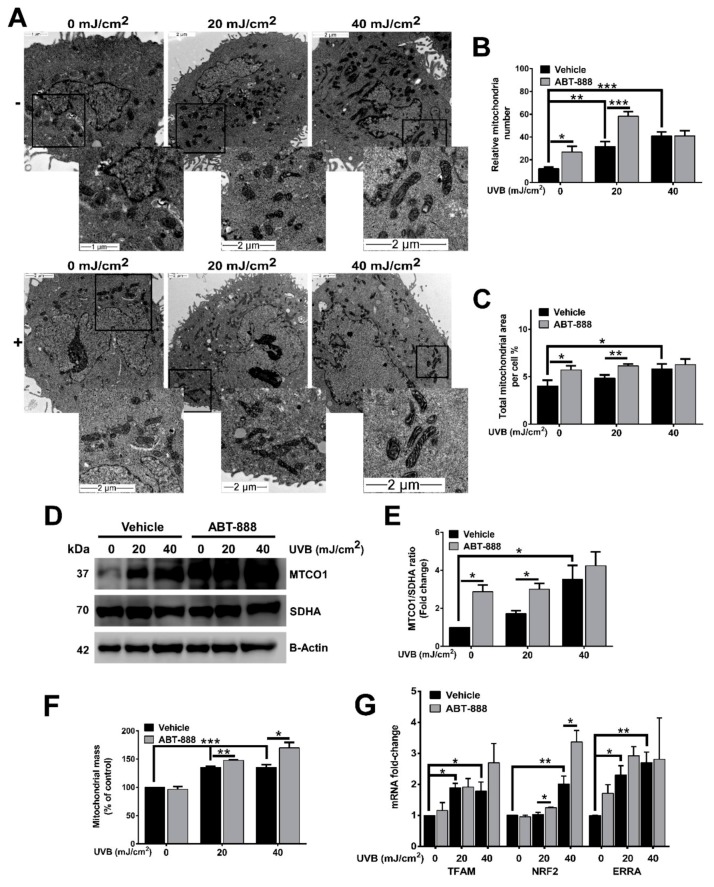
Poly (ADP-ribose) polymerase (PARP) inhibition enhances ultraviolet B (UVB)-mediated mitochondrial biogenesis. (**A**) Effect of UVB irradiation and PARP inhibition on mitochondrial ultrastructure visualized by transmission electron microscopy (TEM) 24 h after UVB exposure. Enlarged pictures are displayed at the right bottom corner. Scale bar is presented on the lower panels. (**B**) Mitochondrial number and (**C**) area were calculated from TEM images (minimum 7 cells were analyzed). (**D**,**E**) Mitochondrial biogenesis was quantified by the ratio of the mitochondrially encoded cytochrome C oxidase I (MTCO1) and succinate dehydrogenase complex, subunit A (SDHA) expression 24 h post UVB (*n* = 3). (**F**) Mitochondrial mass was determined by Mitotracker Green labeling 24 h after UVB irradiation (*n* = 3). (**G**) mRNA levels of master regulators of mitochondrial biogenesis were quantified by real-time PCR 24 h post-UVB (*n* = min. 3). −/+ represent vehicle (−) or ABT-888 (+) treatment. *; ** and *** indicate statistically significant difference at *p* < 0.05 and *p* < 0.01, *p* < 0.001, respectively. Error bars represent SEM.

Mitochondrial biogenesis, by promoting the growth, formation, and assembly of newly synthesized mitochondria, has recently been linked to cancer development [61], apoptosis [62,63,64], and DNA damage [18,28,65]. Accumulating evidence suggest that DNA damage can initiate mitochondrial biogenesis which is accompanied by elevation in mitochondrial number, area, and mass [18,28,65,66]. Transmission electron microscopic images revealed that UVB-treated cells contain more and longer cristae than non-irradiated cells (Figure 3A). This morphological alteration became more pronounced after PARP inhibition. Similarly, UVB treatment increased both mitochondria number and total mitochondrial area (Figure 3B,C). ABT-888 treatment resulted in further increase in these parameters suggesting that PARP inhibition may boost UVB-mediated mitochondrial response. Since mitochondrial content changes with the balance between mitochondrial biogenesis and turnover, we quantified mitochondrially encoded cytochrome C oxidase I (MTCO1)/succinate dehydrogenase complex, subunit A (SDHA) ratio that is a marker of mitochondrial biogenesis. This experiment demonstrated that the mitochondrially-encoded MTCO1 show strong induction after UVB irradiation and become even more expressed after PARPi, while the expression of the nuclearly-encoded SDHA protein is unaltered (Figure 3D,E). The higher mitochondrial mass after both UVB and PARPi (Figure 3F) and the enhanced expression of the master regulators of mitochondrial biogenesis including mitochondrial transcription factor A (TFAM), nuclear respiratory factor 2 (NRF2), and estrogen-related receptor alpha (ERRA) (Figure 3G) also suggest that PARPi augments the UVB-triggered mitochondrial biogenesis.

### 2.3. PARP Inhibition Augments UVB-Mediated Mitochondrial Fusion

**Figure 4:**
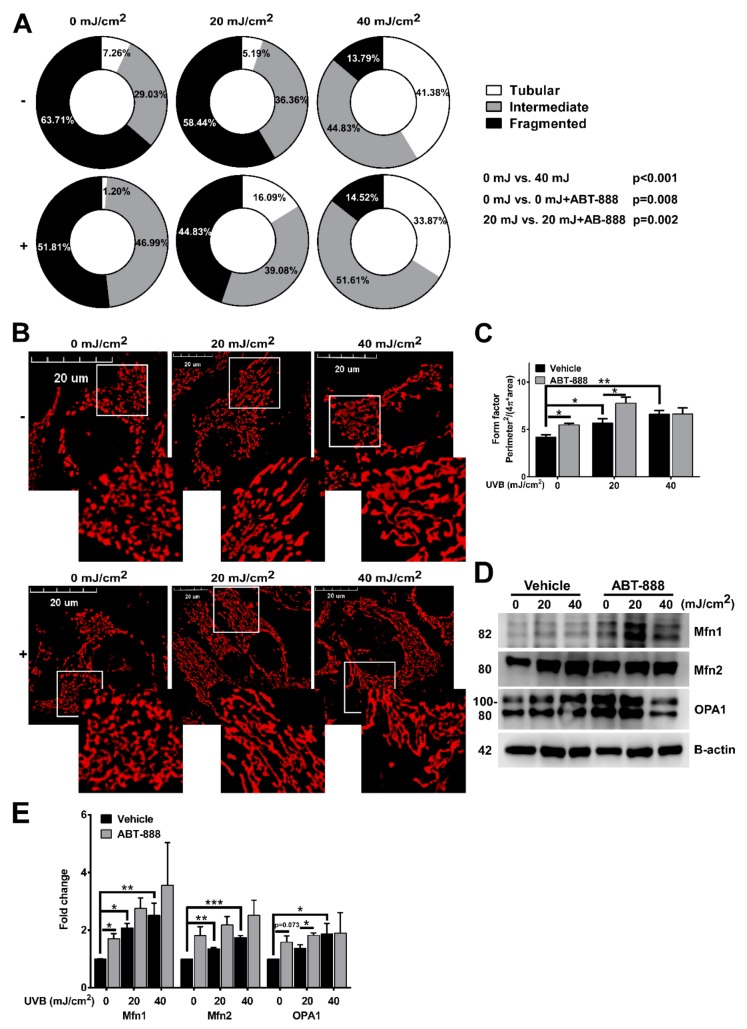
Poly (ADP-ribose) polymerase (PARP) inhibition augments ultraviolet B (UVB)-mediated mitochondrial fusion. (**A**) Effect of UVB irradiation and PARP inhibition on mitochondrial morphological subtypes (tubular, intermediate, and fragmented) are quantified based on confocal microscopic images 24 h post-UVB (minimum 29 cells). (**B**) Mitochondrial morphology visualized by confocal microscopy with Mitotracker Red CMXRos dye (*n* = 3). Enlarged pictures are displayed at the right bottom corner. Scale bar is presented on the images. (**C**) The branching aspect of mitochondria was derived from confocal microscopic images and was represented as a form factor (*n* = 3). (**D**) Protein expression of mitofusin-1 (Mfn1), mitofusin-2 (Mfn2), and optic atrophy 1 (OPA1) was visualized by Western blot. (**E**) Expression of mitochondrial fusion proteins were analyzed by Western blot 24 h post-UVB (*n* = min. 3). −/+ represent vehicle (−) or ABT-888 (+) treatment. *; ** and *** indicate statistically significant difference at *p* < 0.05 and *p* < 0.01, *p* < 0.001, respectively. Error bars represent SEM.

To identify if UVB and PARPi also alters mitochondrial dynamics, we evaluated mitochondrial morphology using confocal microscopy. Non-irradiated cells mainly contained fragmented mitochondria which normally represents low metabolic activity. After 40 mJ/cm^2^ UVB, a statistically significant reduction in the frequency of fragmented mitochondria and an elevation in tubular mitochondria was detected compared to the non-irradiated control. The frequency of intermediate mitochondria was increased after PARPi at 0 mJ UVB compared to the vehicle control, and we also observed decreased fragmented mitochondrial frequency and higher percentage of tubular mitochondria after 20 mJ/cm^2^ + ABT-888 treatment compared to the 20 mJ/cm^2^ UVB exposed cells (Figure 4A). The dose-dependent effect of UVB in the branching aspect of mitochondria (Figure 4C) as defined by form factor ((Perimeter^2^/(4π × area)) was also observed suggesting enhanced mitochondrial fusion after UVB and PARP inhibition. Finally, to confirm that the observed mitochondrial fusion are regulated by the dynamin-related proteins, we checked the expression of mitofusin-1 (Mfn1), mitofusin-2 (Mfn2), and optic atrophy 1 (OPA1) (Figure 4D,E). Similarly to mitochondrial morphological alterations, we detected enhanced protein expression of Mfn1, Mfn2, and OPA1 which support mitochondrial fusion at the outer and inner mitochondrial membrane. These results clearly indicate that besides enhancing mitochondrial biogenesis, UVB also triggers mitochondrial fusion and increases the complexity of mitochondrial network which is more prominent after PARP inhibition.

### 2.4. PARP Inhibition and UVB Induces Bulk Autophagy but Not Mitophagy

**Figure 5:**
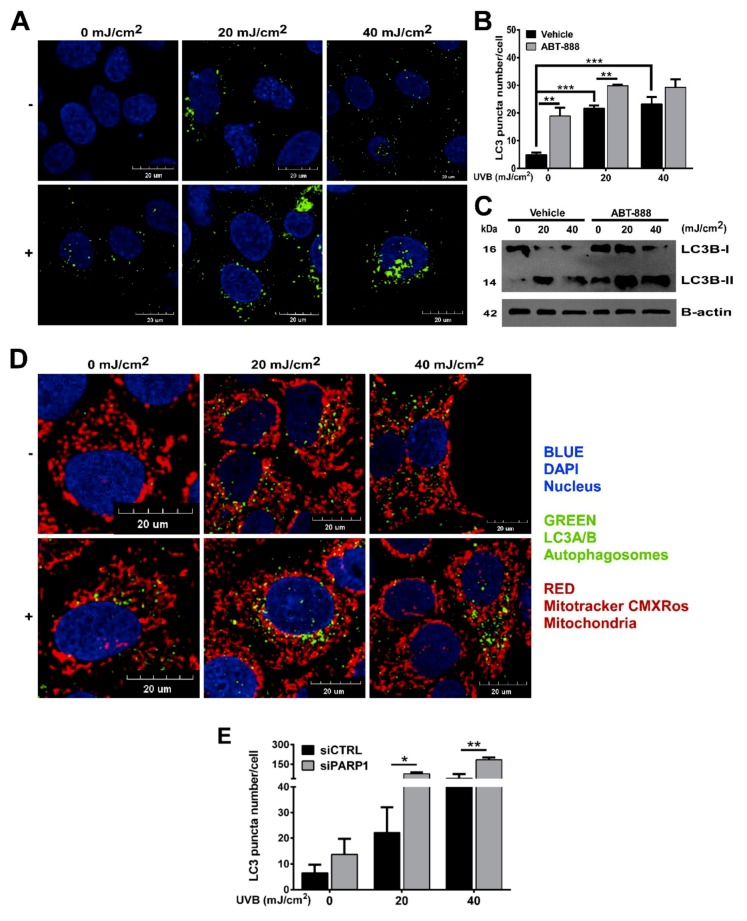
Poly (ADP-ribose) polymerase (PARP) inhibition and ultraviolet B (UVB) induces bulk autophagy but not mitophagy. (**A**) Expression of microtubule-associated proteins 1A/1B light chain 3B (LC3A/B) protein marker of autophagy by confocal microcopy (*n* = 3). Scale bar is presented on the figure. (**B**) Quantification of LC3A/B puncta in cells derived from confocal images in Figure 5A (*n* = 3). (**C**) LC3A/B proteins were quantified by Western blotting. Brightness and contrast were adjusted. Protein of interest were normalized to the loading control B-actin. (**D**) Dual staining of LC3-puncta and mitochondria by confocal microscopy (*n* = 3). (**E**) Quantification of LC3A/B puncta in PARP1 siRNA and control siRNA-transfected cells derived from confocal images (*n* = 4) −/+ represent vehicle (−) or ABT-888 (+) treatment *; ** and *** indicate statistically significant difference at *p* < 0.05 and *p* < 0.01, *p* < 0.001, respectively. Error bars represent SEM.

Several DNA-damaging agents were shown to initiate autophagy [67,68] and mitophagy [69] to remove damaged macromolecules or organelles including mitochondria, which prompted us to explore if UVB and PARPi-induced mitochondrial biogenesis and morphological changes of mitochondria affects autophagy or mitophagy. We used dual labelling of Mitotracker CMxROS to stain mitochondria and microtubule-associated proteins 1A/1B light chain 3B (LC3A/B) antibody to detect autophagosomes. Our results show the accumulation of LC3-positive cells after UVB exposure. PARP inhibition augmented autophagy not only in UVB-exposed cells, but also under non-irradiated conditions (Figure 5A,B). LC3B western blotting (Figure 5C) also confirmed these results indicating the autophagy inducer role of ABT-888. PARP1 knockdown also showed similar results but with much more pronounced autophagy induction after UVB (Figure 5E). Interestingly, the role of PARP1 in the regulation of autophagy is controversial. Both autophagy inductor [70,71,72] and inhibitor [73,74,75] role of PARP1 has been described suggesting that the autophagy-modulatory effect of PARylation might show DNA damage and cell type specificity. Even though, we detected an increased number of LC3 puncta, this type of macroautophagy cannot be considered as mitophagy since autophagic puncta show very mild colocalization with mitochondria (Figure 5D). Furthermore, a dose-dependent mitochondrial elongation was observed after UVB and PARP inhibition in Figure 4A,B indicating mitochondrial fusion. Since fused mitochondria are protected from mitophagy [76], and mitophagy-coupled elimination of mitochondria is usually preceded by fission [69] and decline in mitochondrial function [77], the here experienced induction in autophagy is considered as general autophagy and suggest that mitochondrial morphological rearrangements induced by UVB and PARPi may interfere with the initiation of mitophagy.

### 2.5. PARP Inhibition Boosts UVB-Mediated Mitochondrial Bioenergetic Changes

**Figure 6:**
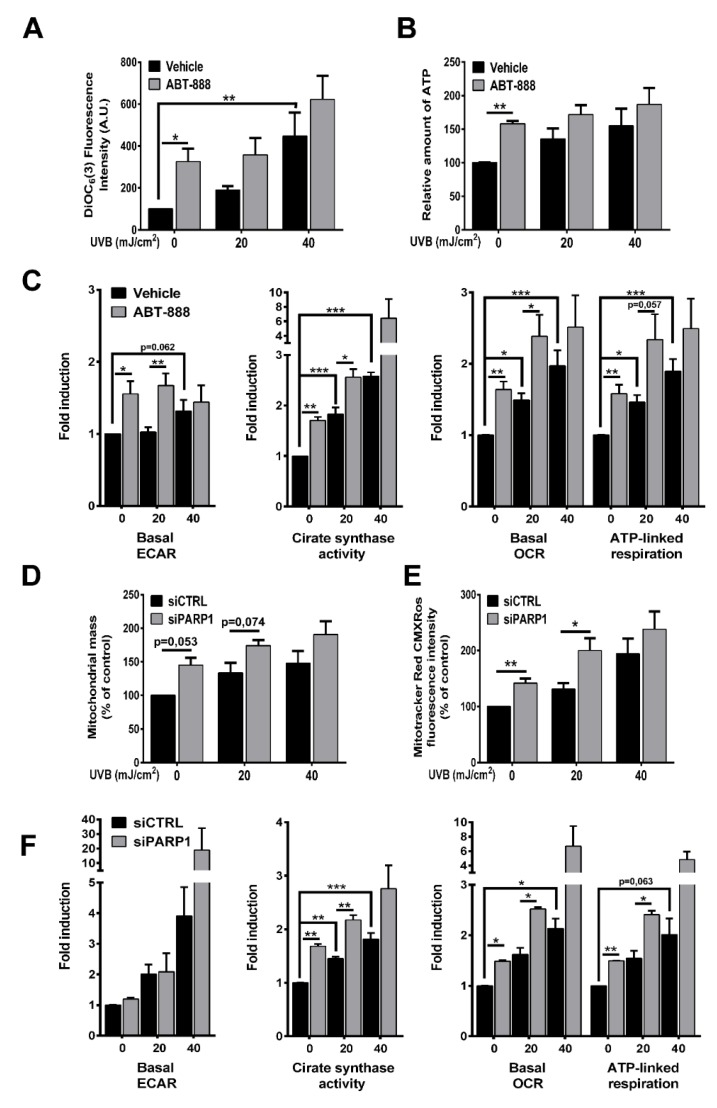
PARP inhibition boosts UVB-mediated mitochondrial bioenergetic changes. (**A**) For determination of mitochondrial membrane potential cells were stained with 3,3′-dihexyloxacarbocyanine iodide (DIOC_6_(3)) 24 h post-UVB and measured by flow cytometry in FL1 channel (*n* = 4). (**B**) Total cellular ATP level was quantified by colorimetric assay (*n* = 3). (**C**) Basal extracellular acidification rate (ECAR) represents glycolysis after 24 h post-UVB. XF medium was supplemented with 10 mM glucose (*n* = 6). After four oxygen consumption rate (OCR) measurement oligomycin and antimycin was used to determine mitochondria-linked ATP production and basal OCR, respectively (*n* = 6). Citrate synthase activity, the initial enzyme of the tricarboxylic acid (TCA) cycle was measured by citrate synthase kit (*n* = 3). (**D**) Mitochondrial mass determined similarly as in Figure 3F (*n* = 4). (**E**) Mitotracker Red CMXRos mean fluorescence intensity was measured by flow cytometry (*n* = 4). (**F**) Metabolic parameters including glycolysis, citrate synthase activity, and oxidative phosphorylation (OPXHOS) was detected similarly as in Figure 6C (*n* = 2 for ECAR and OCR, *n* = 3 for citrate synthase (CS) activity). * *p* < 0.05, ** *p* < 0.01, *** *p* < 0.001.

Alterations in the mitochondrial network is strictly controlled by intra- or extracellular signals connecting mitochondrial biogenesis and fusion with energy perturbations. To test whether increased mitochondrial biogenesis and fusion after UVB and PARP inhibition supports mitochondrial activity, we evaluated mitochondrial parameters. Our results show that UVB exposure increased mitochondrial membrane potential (Figure 6A) and total ATP level (Figure 6B). PARPi boosted the UVB-induced mitochondrial membrane hyperpolarization and it also raised ATP level compared to UVB-irradiated samples. Mitochondrial membrane potential and ATP levels change independently, and hyperpolarization of mitochondrial membrane potential does not necessarily correlate with ATP production but it may stem from decreased F_0_F_1_ ATP synthase activity and concomitant lower ATP production. Furthermore, increased ATP level may result either from enhanced glycolysis, increased respiration via electron flow from complex I-V, or decreased energy expenditure. In order to clarify the reasons of the elevation in ATP levels, we complemented our data with quantitative analysis of metabolic flux by XF96 oximeter and monitored extracellular acidification rate (ECAR) indicating glycolysis and oxygen consumption (OCR) representing oxidative phosphorylation with sequential addition of oligomycin and antimycin. Although basal ECAR (Figure 6C) is statistically unchanged after UVB exposure, PARP inhibition enhanced the rate of glycolysis after 20 mJ/cm^2^ UVB dose and at non-irradiated conditions. In contrast, UVB dose-dependently increased oxygen consumption and ABT-888 treatment significantly augmented OXPHOS compared to 0 or 20 mJ/cm^2^ UVB (Figure 6C). Citrate synthase (CS) activity, the initial enzyme in the tricarboxylic acid (TCA) cycle catalyzing the formation of citrate from oxalacetate and acetyl-CoA, showed significant elevation after UVB radiation, which was augmented by PARP inhibition (Figure 6C). This suggest that not only the distal part of respiratory chain is altered by UVB and PARPi, but also increased terminal oxidation is preceded by elevation in TCA activity as well. To exclude the possibility of the off-target effects of ABT-888, we confirmed the mitochondrial changes by PARP1 silencing. We detected increased mitochondrial mass reflecting mitochondrial biogenesis after UVB in control siRNA-transfected keratinocytes that was elevated by PARP1 knockdown (Figure 6D). Mitotracker Red CMXRos incorporation into mitochondria reflecting mitochondrial membrane potential also showed similar changes similarly after vehicle and ABT-888 treatment (Figure 6E). Although, we could not detect difference in basal ECAR between the control and PARP siRNA-transfected cells, CS activity and OXPHOS showed significant changes (Figure 6F) confirming the pivotal role of PARP1 in the UVB-induced mitochondrial changes. In summary, we can conclude that elevation in cellular ATP level after UVB is due to enhanced energy production and cells increase their energy reserves through both glycolysis, TCA cycle, and OXPHOS after PARP inhibition. 

### 2.6. PARP Inhibition Restores NAD^+^ Level and SIRTUIN Expression

**Figure 7:**
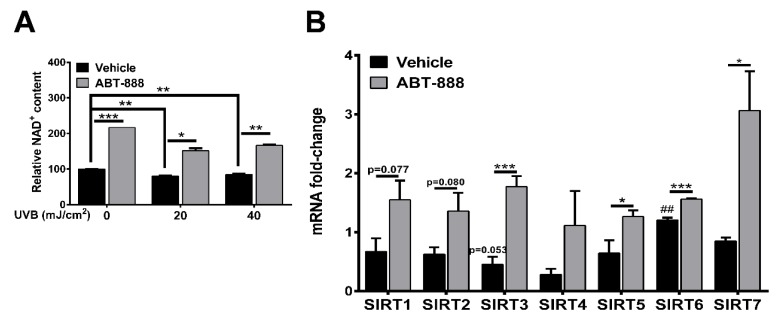
PARP inhibition restores NAD^+^ level and Sirtuin expression. (**A**) Total cellular NAD^+^ were quantified by colorimetric assay 24 h after UVB irradiation. Absorbance was measured at 490 nm (*n* = 2). (**B**) mRNA expression of the Sirtuin enzyme family 24 h post-UVB (40 mJ, *n* = 3). *; ** and *** indicate statistically significant difference at *p* < 0.05 and *p* < 0.01, *p* < 0.001, respectively. ## indicate significant difference at *p* < 0.01 compared to non-irradiated control. Error bars represent SEM.

NAD^+^ and NADH plays a central role in redox homeostasis and cellular metabolism including glycolysis via glyceraldehyde-3-phosphate dehydrogenase (GAPDH) activity [78] and OXPHOS by regulating the transfer of electrons to complex I [79]. UVB irradiation caused a slight but significant decrease in NAD^+^ level after both 20 and 40 mJ/cm^2^ UVB (Figure 7A). ABT-888 treatment efficiently restored intracellular NAD^+^ content suggesting the role of PARP1 in UVB-mediated NAD^+^ depletion. It has been known that a decrease in NAD^+^ by enhanced PARP activation inhibits another NAD^+^-consuming enzyme family, the class III histone deacetylase Sirtuins [33,34,36,37,38,80] which regulate diverse cellular processes [81] including mitochondrial metabolism and have intricate relationships with PARPs [82,83]. To test whether UVB modulate Sirtuin expression and PARPi can restore their expression after UVB similarly as NAD^+^ level changes, we chose the 40 mJ/cm^2^ UVB dose which caused a more significant increase in NAD^+^ level after ABT-888 treatment compared to UVB-irradiated cells. We detected slight mRNA downregulation in SIRT1, SIRT2, SIRT3, SIRT4, SIRT5, and SIRT7 after 40 mJ/cm^2^ UVB dose (Figure 7B) and PARP inhibition increased the gene expression of all Sirtuins suggesting that intracellular NAD^+^ availability after UVB and PARPi may regulate SIRTs expression. Although, the role of Sirtuins in the UVB-mediated cellular response and skin physiology is poorly characterized [84,85,86,87,88], here we revealed the Sirtuin expression-modulatory effect of NAD^+^ after UVB and PARP inhibition. To explore the potential role of NAD^+^ and Sirtuins in supporting glycolysis or oxidative phosphorylation further experiments are needed.

### 2.7. PARP Inhibition Enhances UVB-Mediated Upregulation of Metabolic Proteins

**Figure 8:**
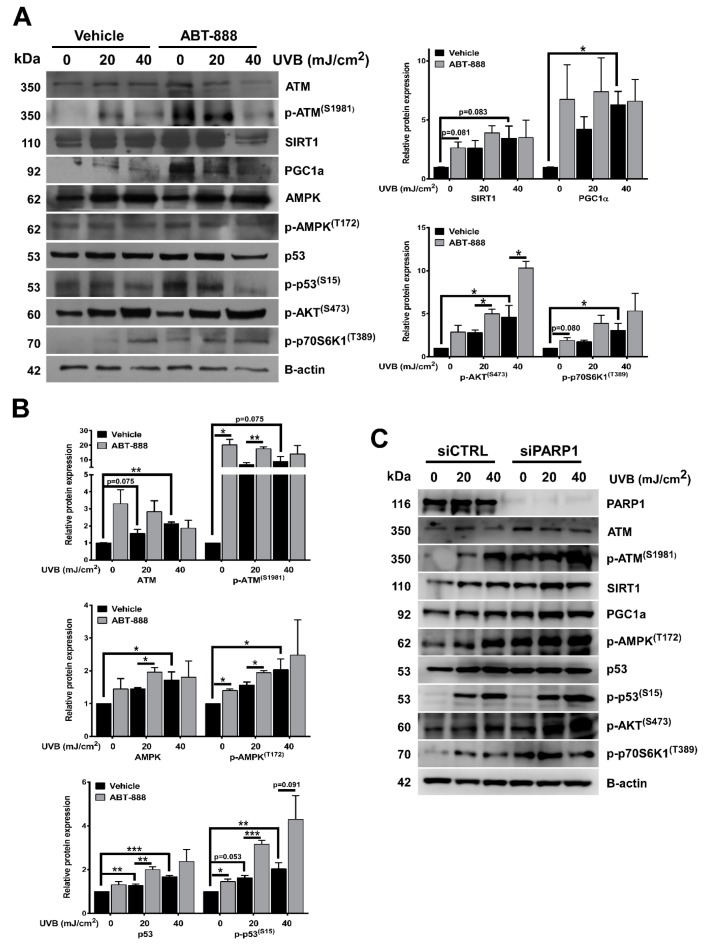
PARP inhibition enhances UVB-mediated upregulation of metabolic proteins. (**A**) Metabolic proteins involved in oxidative phosphorylation were analyzed in total protein lysates. Protein of interest were normalized to the loading control B-actin. Cells were harvested 24 h after UVB. Brightness and contrast were adjusted. (**B**) Densitometric representation of Figure 8A proteins (*n* = min. 3). (**C**) Protein expression involved in oxidative phosphorylation was confirmed by PARP1 knockdown. *; ** and *** indicate statistically significant difference at *p* < 0.05 and *p* < 0.01, *p* < 0.001, respectively. Error bars represent SEM.

To explore the potential mechanisms responsible for elevated oxidative phosphorylation after UVB and PARP inhibition, we checked the expression of metabolic proteins involved in oxidative phosphorylation by Western blot (Figure 8A,B). Consistent with their role in mitochondrial activity, we detected significant increase in ATM, p-ATM, SIRT1, peroxisome proliferator-activated receptor gamma coactivator 1-alpha (PGC1A), AMPK, p-AMPK, p53, p-p53, p-AKT, and p-p70S6K1 (mTOR activity) expression after UVB which was further enhanced by PARP inhibition. The most prominent and statistically significant upregulation in these proteins can be detected after 40 mJ/cm^2^ UVB dose similarly as OXPHOS was increased to two-fold at *p* < 0.001. PARPi induced statistically significant difference at 0 and 20 mJ/cm^2^ compared to vehicle control similarly as ABT-888 increased OXPHOS as well. PARP1 knockdown also induced upregulation in these proteins compared to siRNA control. These results suggest that these proteins switch on oxidative metabolism and their elevated expression may be responsible for higher oxygen consumption after UVB and ABT-888 treatment.

### 2.8. PARP Inhibition and UVB-Induced Oxidative Phosphorylation and Autophagy Are Dependent on ATM, AMPK, p53, AKT, and mTOR Activation

**Figure 9:**
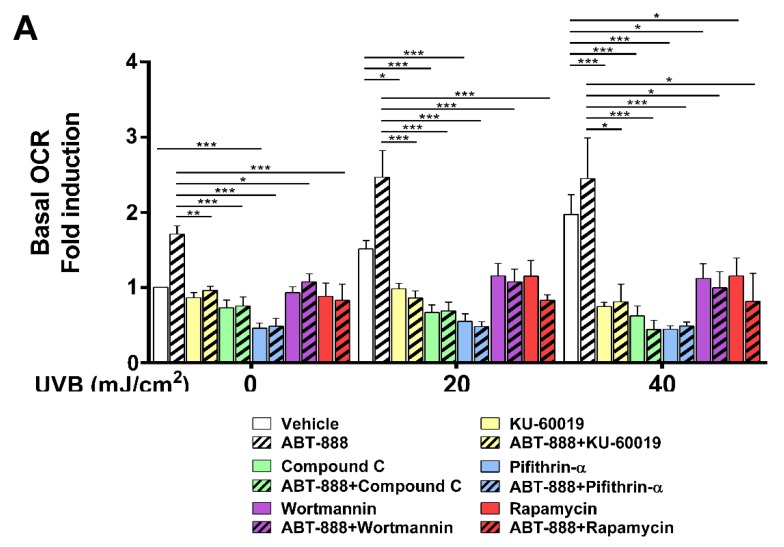

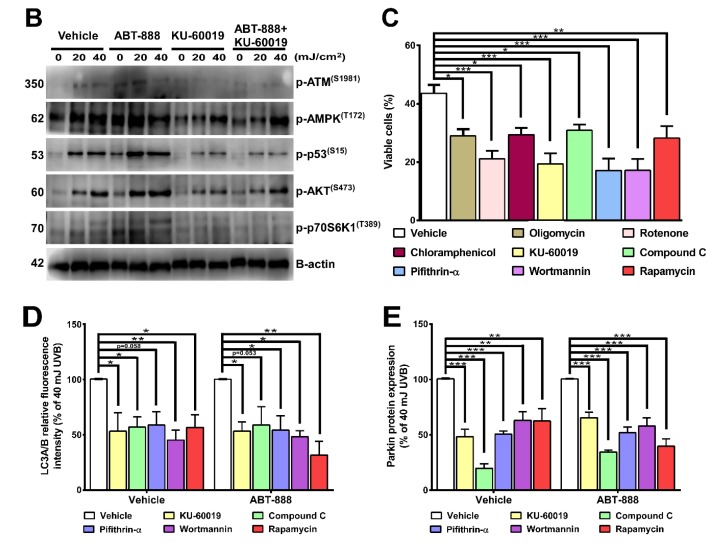
PARP inhibition and UVB-induced oxidative phosphorylation and autophagy are dependent on ATM, AMPK, p53, AKT, and mTOR activation. (**A**) To determine key proteins involved in mediating mitochondrial changes ATMi (KU-60019), AMPKi (Compound C), p53i (Pifithrin-alpha-HBr), PI3Ki (Wortmannin), and mTORi (Rapamycin) were added to the medium and OCR was measured as in Figure 6C (*n* = min.3). (**B**) ATM downstream signaling pathway was investigated by Western blot with the addition of its pharmacological inhibitors KU-60019 (*n* = 3). Brightness and contrast were adjusted. (**C**) Cell viability was measured by flow cytometry as in Figure 1E after 40 mJ/cm^2^ UVB with oligomycin, rotenone, chloramphenicol, ATMi, AMPKi, p53i, PI3Ki, and mTORi (*n* = min. 4). To determine the involvement of ATM, AMPK, p53, AKT, and mTOR in the regulation of (**D**) autophagy and (**E**) PARKIN expression, we applied their respective pharmacological inhibitors after 40 mJ/cm^2^ UVB. (*n* = 4). *; ** and *** indicate statistically significant difference at *p* < 0.05 and *p* < 0.01, *p* < 0.001, respectively. Error bars represent SEM. ATM: ataxia-telangiectasia-mutated kinase, AMPK: adenosine monophosphate-activated kinase, AKT: protein kinase B, mTOR: mammalian target of rapamycin, OCR: oxygen consumption rate.

**Figure 10:**
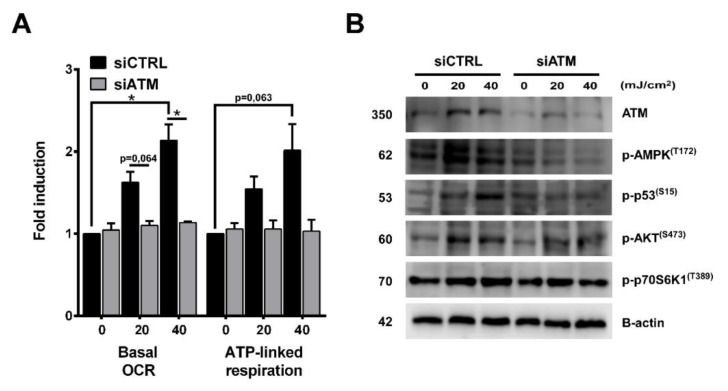
ATM silencing after UVB showed similar results as ATM inhibition by KU-60019. (**A**) OCR was measured as in Figure 6C (*n* = 2) after ATM knockdown. (**B**) ATM downstream signaling pathway was investigated by Western blot with ATM silencing (*n* = 2). * indicates statistically significant difference at *p* < 0.05. Error bars represent SEM.

To test their requirement for increased oxidative phosphorylation, we applied KU-60019 as an ATM inhibitor (Figure 9A) and ATM siRNA for gene silencing (Figure 10A), compound C as an AMPK inhibitor, Pifithrin-alpha-HBr as a p53 inhibitor, Wortmannin as a phosphoinositide 3-kinase (PI3Ki)/AKT inhibitor and Rapamycin as a mTOR inhibitor (Figure 9A). Chemical inhibition of these proteins led to significant reduction in OXPHOS highlighting their role in mediating metabolic alterations after UVB and PARP inhibition. ATM inhibition (Figure 9B) and ATM knockdown (Figure 10B) also decreased the phosphorylation of AMPK, p53, AKT, and p70S6K1 which suggest that elevation in OXPHOS after UVB is downstream of ATM, which is known as one of the most important players in DNA damage response besides PARP1. Since CPD removal is mediated by the energetically demanding nucleotide excision repair, keratinocytes may try to compensate their increased DNA damage with a more intense mitochondrial activity to facilitate CPD elimination. In this respect, OXPHOS seems to be a beneficial response after UVB. To confirm this hypothesis, we applied inhibitors that decreased (ATMi, AMPKi, p53i, PI3Ki, mTORi) or limit the activity of mitochondrial electron transport chain (oligomycin and rotenone) or inhibits mitochondrial protein synthesis (chloramphenicol) (Figure 9C). Our data show that all these inhibitions led to a more pronounced cell death after UVB which confirms that increased mitochondrial activity bears an adaptive and antiapoptotic response after UVB. We also wanted to explore if UVB and PARPi-triggered general autophagy (Figure 5A,B) by recycling damaged organelles and macromolecules serves to provide metabolites for OXPHOS. To confirm this hypothesis, we tested the ATM, AMPK, p53, PI3K, and mTOR inhibitors after 40 mJ/cm^2^ UVB that caused the most prominent decrease in oxygen consumption (Figure 9A). Chemical inhibition of these proteins induced significant reduction in LC3B (Figure 9D) and Parkin (Figure 9E) expression similarly as UVB and PARPi-induced OXPHOS decreased after ATM, AMPK, p53, PI3K, and mTOR inhibitors indicating a parallel reduction in oxidative phosphorylation, autophagy, and PARKIN expression (a marker for mitophagy) as well. Reduction in PARKIN expression suggests impaired mitochondrial quality control, allowing the accumulation of damaged mitochondria, corrupting mitochondrial function, eventually leading to decreased OXPHOS. These results together suggest that after UVB and ABT-888 treatment, AMPK, p53, AKT, and mTOR are the main mediators of oxidative phosphorylation downstream of ATM kinase, and autophagy by recycling damaged cellular parts after stress response may provide metabolites to mitochondria and supports OXPHOS after UVB exposure and PARP inhibition.

## 3. Discussion

In this study, we identified morphological and functional changes of mitochondria after UVB and PARP inhibition. Early after UVB exposure, we detected PAR formation, reflecting PARP activity which was completely suppressed by PARP inhibition. We showed that loss of PARylation caused defective CPD repair compared to the UVB-irradiated cells suggesting impaired NER pathway as several PARP1-intercating partners (DDB1, DDB2, ATM, RAD51, ALC1, XPC) [89,90,91] have been described so far. Furthermore, PARP1 promotes the establishment of locally relaxed chromatin structure [92] to enable the removal of damaged DNA parts. Therefore, it is logical to assume that PARP inhibition renders chromatin to become compact, blocking not only replication, transcription but also the repair of UVB-induced photoproducts suggesting the particular importance of PARP1 in mediating transcription coupled nucleotide excision repair. Our results show a prolongation in G_2_/M phase of cell cycle after PARPi as described earlier [93,94,95]. Inhibition of PARP1 was synergistic with UVB with respect to G_2_/M accumulation characteristic of severe DNA damage after 20 mJ/cm^2^. G_2_/M accumulation can also be attributed to PARP trapping, in which inactivated PARP1 remains bound to DNA stalling replication, transcription leading to replicative stress and double-strand breaks [96,97] which make PARP inhibitors cytotoxic in combination with DNA damaging agents [98,99]. If DNA damage cannot be repaired, cells try to evade the accumulation of mutations by initiating apoptosis. Since apoptotic response after UVB cannot be ameliorated by PARPi, we can exclude the possibility that UVB-triggered cell death in our model system is PARP1-dependent, it may rather suggest that the accumulated and unrepaired CPDs augments the apoptotic response after PARPi. Long-term survival of keratinocytes revealed that the combined treatment of UVB and PARP inhibition severely reduced cell proliferation even at the non-irradiated conditions possibly suggesting the role of trapped PARP1-DNA complexes preventing cells division. We also detected that loss of PARylation decreased the frequency of mutations in UVB-treated cells as evidenced by decreased number of colonies after ABT-888 treatment suggesting that PARPi initiate apoptosis of cells with a high content of CPDs. Our results are in accordance with the anti-cancer effect of diverse PARP inhibitors and provide experimental evidence for the photosensitizing effect of PARP inhibitors experienced clinically [100,101]. Nevertheless, it is important to know that therapeutic application of PARP inhibitors might be associated with photosensitivity (sunburn) but not with an increased photocarcinogenesis risk.

Emerging evidence suggest that autophagy induction can be coupled to DNA damage response [67]. Accordingly, UVB triggered the accumulation of autophagosomes which corroborates with enhanced LC3B expression. PARP inhibition resulted in enhanced autophagosome formation and higher LC3B protein level. The role of PARP1 in the regulation of autophagy is still poorly understood, both autophagy inducer [70] and inhibitory effect of PARP1 [75] can be found in the literature. In our model system, increased autophagosome formation after UVB and PARPi can be attributed to the elevated level of DNA damage (higher CPD content), which serves as a positive signal for autophagy since the initiation of global genome NER (GG-NER) subpathway is controlled by autophagy [67,68].

As Le Brace et al. [28] demonstrated, different DNA damaging agents including UVC which also induce CPD formation as UVB, led to AMPK activation and increased fatty acid oxidation. Similarly to their results, UVB radiation triggered mitochondrial fusion and induced mitochondrial biogenesis, which culminated in enhanced mitochondrial activity and complex metabolic events which were more prominent in PARP-inhibited cells. Increased number of mitochondria and mitochondrial area after UVB irradiation was more robust after PARPi. mRNA expression of mitochondrial biogenesis regulators, increased MTCO1/SDHA ratio, higher mitochondrial mass show similar trends suggesting that elevated mitochondrial content is indeed due to enhanced mitochondrial biogenesis and not defective mitochondrial turnover. By measuring mitochondrial function, we observed that PARP inhibition enhanced the UVB-mediated mitochondrial hyperpolarization, raised cellular ATP level and OXPHOS. Interestingly, we detected neither mitochondrial membrane depolarization nor ATP loss which normally occurs after PARP activation via NAD^+^ resynthesis from ATP [102]. Since intracellular ATP concentration is also a regulator in DNA repair and a key factor in the cell’s decision to die via apoptosis or necrosis [103], it is likely that this elevation in ATP level is required for the energetically costly nucleotide excision repair to remove UVB-induced photoproducts, and/or necessary for the apoptotic process as seen after staurosporine treatment [104]. Boost in mitochondrial OXPHOS was dependent on ATM, AMPK, p53, AKT, and mTOR phosphorylation since inhibition of them lead to significant reduction in oxygen consumption after both UVB and PARPi. Moreover, enhanced mitochondrial activity seems to induce a beneficial response after DNA damage, since decreasing mitochondrial biogenesis or ameliorating OXPHOS resulted in elevated cell death after UVB irradiation suggesting the protective effect of OXPHOS on cell viability. Inhibition of the ATM, AMPK, p53, PI3K, and mTOR pathways caused significant reduction in LC3A/B and Parkin expression emphasizing their role in autophagy and mitophagy. Decrease in mitophagy and the concomitant impaired mitochondrial quality control may also be an underlying cause for decreased OXPHOS after chemical inhibition of these proteins. We hypothesize that the purpose of autophagy induction after UVB-induced DNA damage is to recycle damaged molecules by fueling mitochondria with metabolites for oxidative phosphorylation as suspected by Dong et al. [105] and autophagy promotes the DNA damage recognition via NER. Since autophagy is an ATP-dependent process and cells must maintain their energy status to promote autophagy, we cannot exclude reciprocal regulation that is the shift toward oxidative metabolism regulates autophagy as described by Thomas et al. [106]. Nonetheless, in our model system it seems obvious that the purpose of autophagy and OXPHOS induction is to promote cell survival after UVB via ATM, AMPK, p53, AKT, and mTOR activation.

So how, increased mitochondrial activity is connected to DNA damage? The UVB-induced photolesions trigger local conformational changes on DNA similarly as seen after etoposide treatment [107] and UVC irradiation [108] which also resulted in increased mitochondrial biogenesis and activity [18,28]. Since, PARP-inhibited cells displayed higher level of CPDs compared to UVB-irradiated samples, it seems plausible that CPDs generated on DNA may be a trigger for mitochondrial changes. This is supported by the fact that the phosphorylation status of the main DNA damage responders (ATM, AMPK, p53, AKT, and mTOR) that are activated by UVB become more prominent after PARPi. ATM is mainly activated by DNA double-strand breaks but also a sensor of CPDs [109]. Furthermore, unrepaired CPD lesions in PARPi cells due to PARP trapping may eventually be converted into DNA breaks during replication which is an ultimate trigger for ATM activation. ATM can directly phosphorylate AMPK [110], p53 [111], and AKT [112] and via AKT and AMPK signaling ATM regulates mTOR activity [113], as well to turn on oxidative metabolism. AMPK can also phosphorylate p53 at S15 [114], thereby potentiating its activity in enhancing mitochondrial metabolism. Furthermore, it is noteworthy that although cell type-dependently, AMPK [115], p53 [116] AKT [117], and mTOR [118] activation can trigger mitochondrial fusion which may also be a possible explanation for enhanced mitochondrial activity. Since PARP inhibition alone do not induce CPD formation, we must take into account that the PARPi-mediated changes in the non-irradiated cells are mediated by CPD-independent mechanisms either through autophagy induction and/or NAD^+^ prevention. However, we can exclude the involvement of NAD^+^ in the regulation of mitochondrial function after UVB, since morphological alteration of mitochondria and oxidative phosphorylation do not show similar changes as intracellular NAD^+^ availability after UVB irradiation.

## 4. Materials and Methods

### 4.1. Chemicals

All chemicals were obtained from Sigma-Aldrich (St. Louis, MO, USA) unless stated otherwise.

### 4.2. Cell Culture

Human immortalized keratinocyte-derived (HaCaT) cell line were cultured in a T75 flask, as previously described [119] using 4500 mg/L Dulbecco’s modified eagle media (DMEM) Glutamax supplement (Thermo Fisher Scientific, Waltham, MA, USA) containing 10% heat-inactivated fetal bovine serum (FBS) and 0.5% antibiotic/antimycotic solution. For HPRT mutation assay, Chinese hamster ovary (CHO) cells were used as described in Section 4.7. Cells were maintained in a humidified incubator at 37 °C with 5% CO_2_ atmosphere.

### 4.3. Cell Treatment

Cells were harvested with trypsin-EDTA (Biosera, Budapest, Hungary) then seeded in 12-well plate in 200,000 cell/well density (unless stated otherwise), and allowed to adhere for at least 12 h. At 80% confluence, cells were pretreated with the PARP inhibitor veliparib (ABT-888) (Selleckchem, Houston, TX, USA) at a final concentration of 25 µM. For detection of cell death, autophagy, mitophagy, and OXPHOS, the following inhibitors were used: ATMi: 6 µM KU-60019 (Adooq, Irvine, CA, USA), AMPKi: 2.5 µM compound C (Selleckchem), p53i: 30 µM pifithrin-alpha-HBr (AdooQ), mTORi: 300 nM rapamycin (AdooQ), PI3Ki: 300 nM wortmannin (AdooQ), mitochondrial activity inhibitors: 5 µM oligomycin, and 500 nM rotenone. To inhibit mitochondrial protein synthesis and hamper mitochondrial function chloramphenicol (AdooQ) (25 µM) was used. For UVB irradiation, cells were covered with 400 µl pre-warmed Dulbecco’s phosphate-buffered saline (DPBS) (Lonza, Walkersville, MD, USA) and were subjected to 20 or 40 mJ/cm^2^ UVB using two UVB broadband tubes (TL-20W/12 RS; Philips, Eindhoven, The Netherlands). Immediately after irradiation, the old medium was placed back on the cells to evade metabolic perturbations and cells were further cultured for 24 h. Controls were covered with tin foil during irradiation. The proper UV dose was measured with a UVX digital radiometer (UVP Inc., San Gabriel, CA, USA). Detached, dead cells were excluded from the experiments except for cell viability.

### 4.4. Gene Silencing

On-target plus SMARTpool PARP1 and ATM siRNA sequence was purchased from Dharmacon Research, Inc. (Lafayette, CO). Non-targeting siRNA (Dharmacon) was used as a control. HaCaT cells were seeded into 12-well plates in complete high-glucose DMEM containing 10% FBS without antibiotics. DharmaFECT transfection reagent (Dharmacon) in tube 1 and siRNA in 1 × siRNA buffer (Dharmacon) in tube 2 was diluted in serum and antibiotics-free DMEM and incubated at room temperature for 5 min. The two mixtures were combined and incubated further for 20 min and added to the cells at a final siRNA concentration of 50 nM. After 48 h, cells were washed with DPBS, irradiated with UVB, and the medium was replaced with a complete growth medium. Silencing efficiency was determined by Western blotting 24 h post-UVB.

### 4.5. Cell Viability and Proliferation

Over the 24 h period following UVB exposure cell viability was determined by dead cell apoptosis kit containing propidium iodide/Alexa Fluor 488-conjugated Annexin V (Invitrogen, Carlsbad, CA, USA) according to the manufacturer’s instruction. Labelled cells were analyzed by flow cytometry with a FACSCalibur (Becton Dickinson, San Jose, CA, USA) measuring the fluorescence emission in FL1 (530 nm) and FL3 (>575 nm). Double negative cells represent viable cells. For data collection and evaluation CellQuest software 5.2 (Becton Dickinson) and Flowjo single cell analysis software were used.

Cell proliferation was determined by clonogenic assay. Cells were seeded in 100 mm Petri dish at 5000 cells/dish and were allowed to grow for 10 days. The medium was replaced each day to monitor the long-term effect of cell treatments. Ten days later, cells were washed with PBS, fixed with 100% methanol, and stained with May-Grünwald-Giemsa solution (Histolab Products, Västra Frölunda, Sweden).

### 4.6. Cell Cycle Analysis

Cell cycle progression was quantified using propidium iodide (PI) staining. Briefly, cells were trypsinized, fixed with ice-cold 96% ethanol for 10 min, washed twice with PBS, and permeabilized with 0.1% Triton X-100. After extensive washing, cells were incubated in PBS solution containing 0.5 mg/mL RNase at 37 °C for 1 h and counted for cell number normalization. For cell staining, PI was used a final concentration of 20 µg/mL. Unbound PI was eliminated by washing with PBS and doublet discrimination was performed. To determine DNA content samples were analyzed on the x-axis in FL2-A channel using a FACSCalibur flow cytometer.

### 4.7. HPRT Mutation Assay

CHO cells were cultured in a HAT (hypoxanthine–aminopterin–thymidine) medium in T75 flask for one week to remove cells with pre-existing HPRT mutation. After a week, HAT medium was replaced with complete DMEM and cells were allowed to recover for three days. Thereafter, CHO cells were seeded into a 6-well plate and allowed to adhere for 24 h. Next day, cells were cultured with or without 25 µM ABT-888 and UVB irradiation was carried out with 10 or 20 mJ/cm^2^ dose. CHO cells were cultured further for one week in DMEM with sub-culturing three times a week. After a week, cells were trypsinized, counted, distributed to 100 mm Petri dish at concentration of 5 × 10^4^ per Petri dish in selection medium supplemented with 5 µg/mL 6-thioguanine (6-TG), and incubated for 10 days. After 10 days, cells were washed with PBS, fixed with 100% methanol for 10 min, and stained with May-Grünwald-Giemsa solution. Mutant colonies were counted.

### 4.8. CPD-Specific Enzyme-Linked Immunosorbent Assay (ELISA)

Genomic DNA was extracted from HaCaT cells using a Purelink Genomic DNA mini kit (Thermo Fisher Scientific) according to the manufacturer’s instruction. For quantitative detection of CPDs, direct ELISA was applied as previously described [120].

### 4.9. Real-Time Quantitative RT-PCR

Total RNA was isolated using guanidinium isothiocyanate-phenol-chloroform extraction (TRI reagent) (MRC, Cincinnati, OH, USA) according to the protocol by Chomczynski et al. [121]. RNA concentration and purity were determined spectrophotometrically. RNA was deprived of DNA contamination by DNase I treatment (Fermentas, St. Leon-Rot, Germany). Reverse transcription (RT) was carried out using the high-capacity cDNA reverse transcription kit (Applied Biosystems, Foster City, CA, USA) according to the manufacturer’s instruction. Real-time RT-PCR analysis was performed by the SYBR green method using a Lightcyler 480 II (Roche Diagnostics). Reactions were carried out in 384-well optical plates. RNA expression values were determined by 1.8^−ΔCT^ method and normalized for the housekeeping gene SDHA and phosphoglycerate kinase 1 (PGK1) [122]. Primer pairs and corresponding sequences are listed in Supplementary Table S1 “Primer pairs used in the study”.

### 4.10. Western Blot

HaCaT cells were pelleted by mild centrifugation at 1500 rpm for 5 min at 4 °C. The pellets were lysed on ice with RIPA buffer supplemented with a protease inhibitor cocktail in 1:1000. After 5 min incubation on ice, supernatants were obtained by centrifugation of the lysates at 15,000 rpm for 10 min at 4 °C. The concentration of proteins was determined using Pierce BCA assay kit (Thermo Fisher Scientific). The lysates were mixed with 5X loading buffer (Bromophenol blue (0.25%), β-Mercaptoethanol (5%), Glycerol (50%), SDS (sodium dodecyl sulfate; 10%), Tris-HCl (0.25 M, pH 6.8)), boiled for 10 min at 100 °C and subjected to 7.5%, 10%, or 12.5% SDS polyacrylamide gel electrophoresis. Proteins were transferred onto nitrocellulose membrane (Bio-Rad, Hercules, CA, USA). The membranes were blocked in TBST (0.05% Tween 20 in TBS buffer) containing 5% bovine serum albumin (BSA). The incubation with primary antibodies was carried out overnight at 4 °C followed by washing. Horseradish peroxidase (HRP)-conjugated goat anti-mouse or anti-rabbit IgG was used as a secondary antibody (Bio-Rad) at room temperature for 1 h. Proteins were visualized by ECL Prime Western blotting detection reagent (Thermo Fisher Scientific). All the antibodies used for Western blotting were listed in Supplementary Table S2 “Primer antibodies used in the study”. Bands were quantified using the ImageJ open source software [123] (version 1.51k, National Institutes of Health, Bethesda, MD). Proteins of interest were normalized for β-actin. Sample uncut blots are provided on Supplementary Figure S2.

### 4.11. Mitochondrial Mass

24 h post-UVB, Mitotracker Green (Thermo Fisher Scientific) were added to the culture medium at a final concentration of 100 nM and cells were incubated at 37 °C for 30 min. Following incubation, cells were washed with PBS and harvested by trypsinization and then placed on ice. The fluorescence intensity of cells stained with Mitotracker Green was analyzed in a FL1 channel using flow cytometry.

### 4.12. Determination of Mitochondrial Membrane Potential

HaCaT cells were seeded in a culture plate and next day subjected to UVB. 24 h post-UVB, 3,3′-dihexyloxacarbocyanine iodide (DiOC_6_(3)) (Invitrogen) were added to the culture medium at a final concentration of 40 nM and cells were further incubated at 37 °C for 30 min. Following incubation, cells were washed with PBS and harvested by trypsinization and then placed on ice. The fluorescence intensity of cells stained with DiOC_6_(3) was analyzed in FL1 channel using flow cytometry.

### 4.13. Determination of Mitochondrial Ultrastructure, Mitochondrial Number, and Area by Transmission Electron Microscopy (TEM)

After cell treatment, cells were washed with DPBS, harvested by trypsinization, pelleted, and fixed by 3% glutaraldehyde (EMS, Hatfield, PA, USA) in 0.1 M cacodylic acid (EMS) buffer complemented by 5% sacharose for 2 h. After washing steps in 0.1 M cacodylic acid buffer, cells were osmificated in 1% OsO_4_, dehydrated in ascending alcohol row, namely 50% for 2 × 10’, 70% for 2 × 10’, 96% for 2 × 15’, absolute ethanol for 3 × 20’, and propylene oxide for 2 × 10’. Then, overnight a durcupan araldite treatment was used for embedding the samples. Encapsulation occurred in an incubator for 48 h and ultrathin section was made by Leica EM UC7 ultramicrotome (Leica Microsystems, GmbH, Wetzlar, Germany). Standard contrasting was performed by uranyl acetate (EMS) and Reynolds’ lead citrate solution. High resolution TEM images was made by the Jeol JEM 1010 electron microscope and software (JEOL Inc. Peabody, MA, USA).

### 4.14. Assessment of Mitochondrial Morphology and Autophagy by Confocal Microscopy

Cells were plated on glass slides in a 24-well plate and 24 h post-UVB cells were stained with 100 nM Mitotracker Red CMXRos (Thermo Fisher Scientific) dye at 37 °C for 30 min. Cells were washed with PBS, fixed with 3.7% paraformaldehyde solution at room temperature for 10 min and permeabilized with 0.2% Triton X-100 for 10 min. After washing with PBS, blocking was performed by 1% BSA-containing PBS at 37 °C for 1 h. Cells were incubated with an Alexa Fluor 488-conjugated LC3A/B antibody diluted in 1% BSA (1:50) at 4 °C overnight in a humid chamber. Next day, slides were washed three times with PBS. Prior to imaging, cells were stained with a mounting medium with DAPI and analyzed by confocal microscopy using 60x oil immersion objective. Background subtraction, noise reduction, local contrast enhancement, unsharp mask, and bandpass filtering were applied on raw images for better image quality and proper image evaluation [124]. Processed images were analyzed by ImageJ software. Mitochondrial complexity was calculated from confocal microscopic images and was defined as a form factor ((Perimeter^2^/(4π*area)). For flow cytometric analysis of autophagy, cells were washed with PBS and harvested by trypsinization. Fixation, permeabilization, and blocking were carried out as described above. Cells were incubated with Alexa Fluor 488-conjugated LC3A/B antibody using 1:500 dilution at 4 °C overnight. Next day, after extensive washing, cells were analyzed for Alexa-488 fluorescence intensity in FL1 channel by flow cytometry.

### 4.15. Measurement of Citrate Synthase Activity

For determination of citrate synthase activity, a citrate synthase assay kit was used according to the manufacturer’s instruction. Optical density changes by kinetic program was determined at 412 nm using microplate reader. Values were normalized to total protein concentration.

### 4.16. Analysis of Oxygen Consumption and Extracellular Acidification

Oxygen consumption (OCR) and extracellular acidification rate (ECAR) was measured using an XF96 extracellular flux analyzer (Seahorse Bioscience, North Billerica, MA, USA). The rate of oxygen consumption indicates oxidative phosphorylation, whereas ECAR represent lactic acid formed during glycolysis. Cells were seeded in a XF96 cell culture plate at 10,000 cell/well density. One hour prior to the assay, culture medium was replaced with unbuffered DMEM (Seahorse Bioscience) supplemented with 10 mM glucose and then cells were equilibrated in a CO_2_-free incubator for 1 h. After four measurements of oxygen consumption, oligomycin and antimycin A were subsequently injected to determine mitochondria-linked ATP production and basal OCR, respectively. All OCR and ECAR values were normalized to the total protein obtained from cells lysed by 1 M NaOH.

### 4.17. Determination of NAD^+^ Level

The colorimetric assay for NAD^+^/NADH ratio determination was purchased from Biovision (Mountain View, CA, USA). Cells were washed with PBS two times and harvested by a trypsin-EDTA solution and pelleted by mild centrifugation. The pellet was extracted with NAD^+^ or NADH extraction buffer and exposed to freeze/thaw cycles. Samples were spun and the supernatant was transferred to a 96-well plate for NAD^+^/NADH assays. For NAD^+^ determination, half of the samples were transferred into another tube and NAD^+^ was decomposed by heating samples at 60 °C. Finally, working reagent was added to the samples, and optical density was determined at 450 nm. Optical density of the standard curve and samples was used to calculate NAD^+^ content. Values were normalized to total protein concentration.

### 4.18. Measurement of ATP Content

For determination of ATP level, an ATP colorimetric/fluorometric assay purchased from Biovision was used according to the manufacturer. Optical density was determined at 570 nm using a microplate reader. Values were normalized to total protein concentration.

### 4.19. Statistical Analysis

Statistical analysis was carried out by the Kolmogorov–Smirnov test to assess the normality of the population. The frequency of mitochondrial morphological subtypes (fragmented, intermediate, and tubular) was calculated by chi^2^ and Ficher’s exact test. To assess the statistical significance between untreated and differently treated groups, ANOVA complemented by Dunnett’s post-hoc test was used. The comparison of two groups was applied by an independent *t*-test. All data are reported as mean ± SEM. A *p* < 0.05 was considered statistically significant. Statistical significance was determined by the GraphPad Prism 7 (GraphPad Software Inc., San Diego, CA, USA) and SPSS 25 software. (SPSS package for Windows, Release 25.; SPSS, Chicago, IL, USA).

## 5. Conclusions

Our results provide new information about the role of ATM and PARP1 proteins in the regulation of mitochondrial morphology and function after UVB irradiation. Moreover, it suggests that UVB and PARPi-caused elevation of oxidative phosphorylation is mediated by the complex interplay of metabolic sensors including ATM, AMPK, p53, AKT, and mTOR which are the main mediators that connect DNA damage with oxidative metabolism and autophagy.

## Data Availability

All primary data is uploaded to https://figshare.com/s/49fbcd8cfd5802ea15f9 (DOI: 10.6084/m9.figshare.8107727).

## Supplementary Materials

The following are available online at https://www.mdpi.com/2072-6694/12/1/5/s1, Figure S1: Uncut PAR (10H) Western blot, Figure S2: Sample uncut blots, Table S1: Primer pairs used in the study, Table S2: Primary antibodies used in the study.

## Abbreviations

CPD	cyclobutane pyrimidine dimer
PAR	poly (ADP-ribose) polymer
PARP1	poly (ADP-ribose) polymerase 1
ATM kinase	ataxia-telangiectasia-mutated kinase
AMPK	adenosine monophosphate-activated kinase
mTOR	mammalian target of rapamycin
p70S6K1	ribosomal protein S6 kinase beta-1
HPRT	hypoxanthine-guanine phosphoribosyltransferase
PGC1A	peroxisome proliferator activated receptor gamma coactivator-1 alpha
MTCO1	mitochondrially encoded cytochrome C oxidase I
CS	citrate synthase
TCA	tricarboxylic acid
SDHA	succinate dehydrogenase complex, subunit A
PGK1	phosphoglycerate kinase 1
NRF2	nuclear respiratory factor 2
ERRA	estrogen-related receptor alpha
Tfam	mitochondrial transcription factor A
OCR	oxygen consumption rate
ECAR	extracellular acidification rate
OXPHOS	oxidative phosphorylation
CHO	Chinese hamster ovary
Mfn1	mitofusin 1
Mfn2	mitofusin 2
OPA1	optic atrophy 1
LC3A/B	microtubule-associated proteins 1A/1B light chain 3B
GAPDH	glyceraldehyde-3-phosphate dehydrogenase
PI3K	phosphoinositide 3-kinase
DiOC_6_(3)	3,3′-dihexyloxacarbocyanine iodide
