# Supplementary material for: PARP1 Inhibition Augments UVB-Mediated Mitochondrial Changes—Implications for UV-Induced DNA Repair and Photocarcinogenesis
Source: Cancers (Basel). 2019 Dec 18;12(1):5. doi: 10.3390/cancers12010005 (PMC7016756; doi:10.3390/cancers12010005)
Supplement: Supplementary file 1 [file cancers-12-00005-s001.pdf]

Supplementary Materials

Table S1. Primer pairs used in the study.

Gene Name	Forward Primer (5′-3′)	Reverse Primer (5′-3′)
SDHA	5′-TGGGAACAAGAGGGCATCTG-3′	5′-CCACCACTGCATCAAATTCATG-3′
PGK1	5′-GCGGGTCGTTATGAGAGT-3′	5′-CTCCATTGTCCAAGCAGAAT-3′
NRF2	5′-TGGCACAAAACACATCACAAC-3′	5′-CTTCATTACCCAAACCACCCA-3′
TFAM	5′-AGCTCAGAACCCAGATGCAAAAA-3′	5′-TACCTGCCACTCCGCCCTATAA-3′
ERRA	5′-GCCACAAGGAAGAGGAGGATG-3′	5′- TCACAGGATGCCACACCATAG-3′
SIRT1	5′-CCCTCAAAGTAAGACCAGTAG-3′	5′-AGATGAGGCAAAGGTTCTCT-3′
SIRT2	5′-AGCCAACCATCTGTCACTACTTC-3′	5′-CGCCTCCACCAAGTCCTC-3′
SIRT3	5′-CAGAGGGTGGTGGTCAT-3′	5′-GGGTTGTGAAAGAAGAATGGGA-3′
SIRT4	5′-CACTTGAATCTTGCTGCTAAATG-3′	5′- TGGTTGAATGGGAACTGGAATCT-3′
SIRT5	5′-ATGGGGTAGGGGTAAGGT-3′	5′-ATCGCTGTGTTTCTCTCCTC-3′
SIRT6	5′-GAAGCCACACCCCAGAG-3′	5′-ACAACACAGCAAGTCAGAG-3′
SIRT7	5′-GCCAACCCTCACCCACAT-3′	5′-TGGAGCCCGTCACAGTTC-3′

Table S2. Primary antibodies used in the study.

Antibody	Dilution	Vendor
anti-PAR (10H)	1:500	Santa Cruz
ATM (D2E2)	1:1000	Cell Signaling Technology
phospho-ATM^{S1981}	1:1000	Cell Signaling Technology
AMPKα (23A3)	1:1000	Cell Signaling Technology
phospho-AMPKα^{T172} (40H9)	1:1000	Cell Signaling Technology
p53 CM 042 C	1:1000	Biocare Medical
phospho-p53^{S15} (16G8)	1:1000	Cell Signaling Technology
phospho-AKT^{S473} (D9E) XP®	1:1000	Cell Signaling Technology
phospho-p70S6K1^{T389} (108D2)	1:1000	Cell Signaling Technology
LC3A/B (D3U4C) XP®	1:1000	Cell Signaling Technology
PGC1A (ab106814)	1:1000	Abcam
SIRT1 07-131	1:1000	Millipore
Mfn1 (D6E2S)	1:1000	Cell Signaling Technology
Mfn2 (D1E9)	1:1000	Cell Signaling Technology
OPA1 (D6U6N)	1:1000	Cell Signaling Technology
β-actin (8H10D10)	1:4000	Cell Signaling Technology
SDHA (PA5-79962)	1:1000	ThermoFisher Scientific
MTCO1 (PA5-79701)	1:500	ThermoFisher Scientific
Parkin (PA5-13399)	1:1000	ThermoFisher Scientific

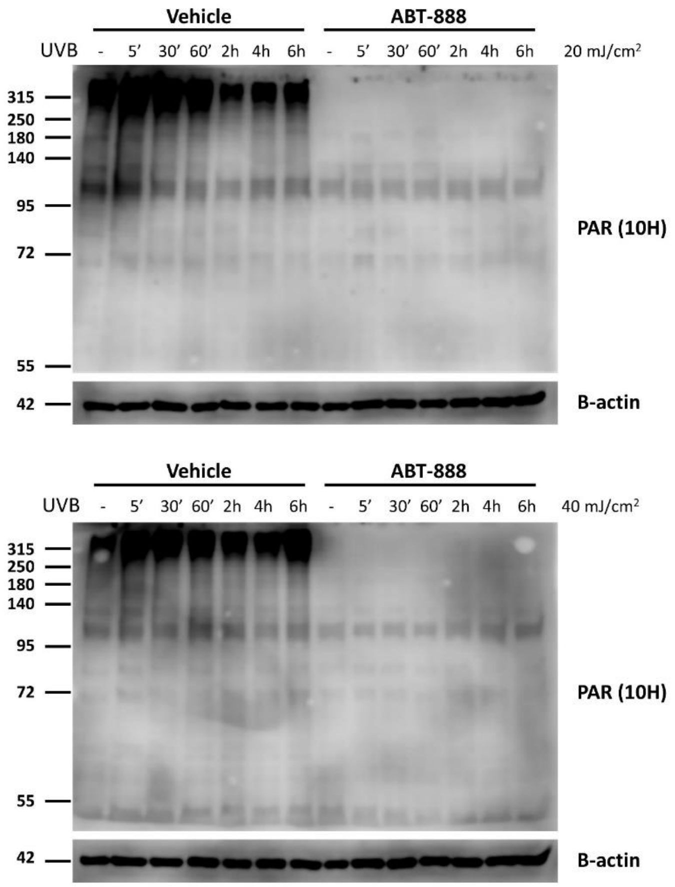

Figure S1. Uncut PAR (10H) Western blot.

Figure 3. western blots

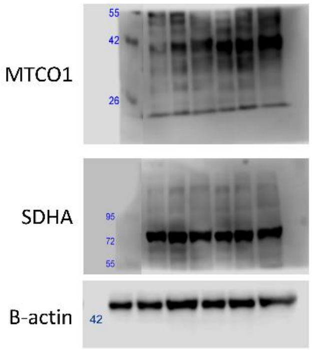

Figure 4. western blots

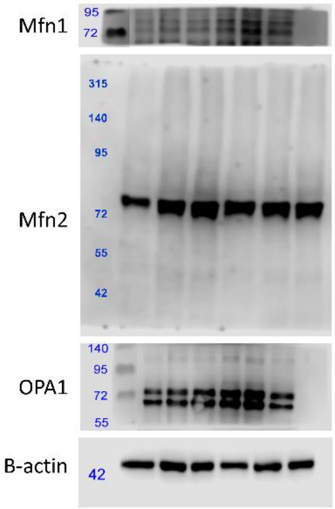

Figure 5. western blots

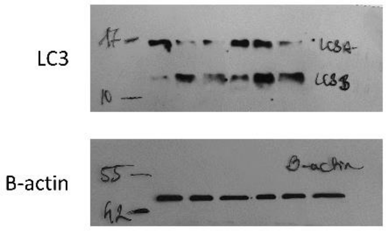

Figure 8. western blots (PARP1 inhibition)

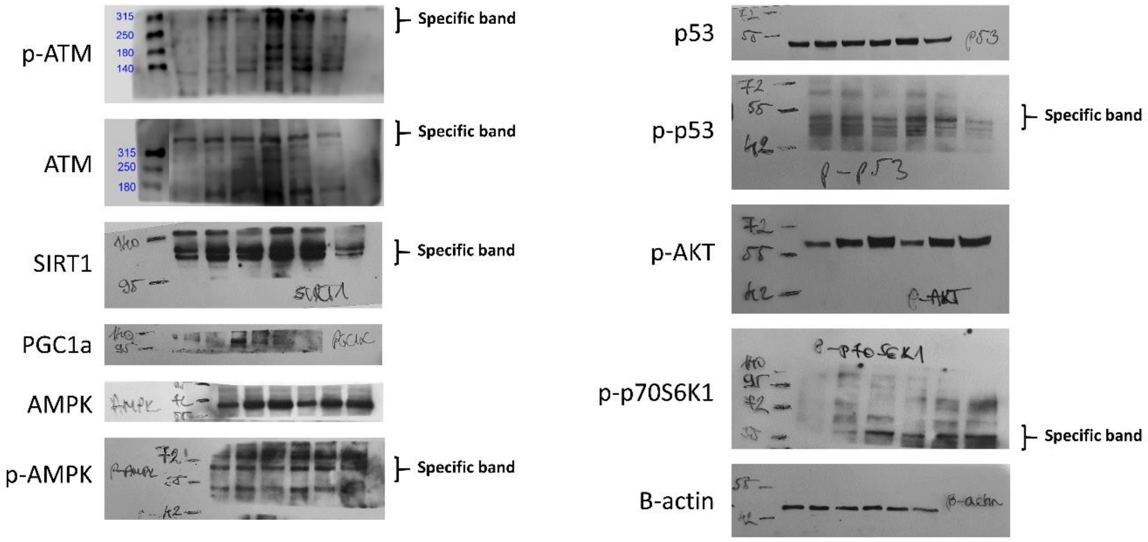

Figure 8. western blots (PARP1 knockdown)

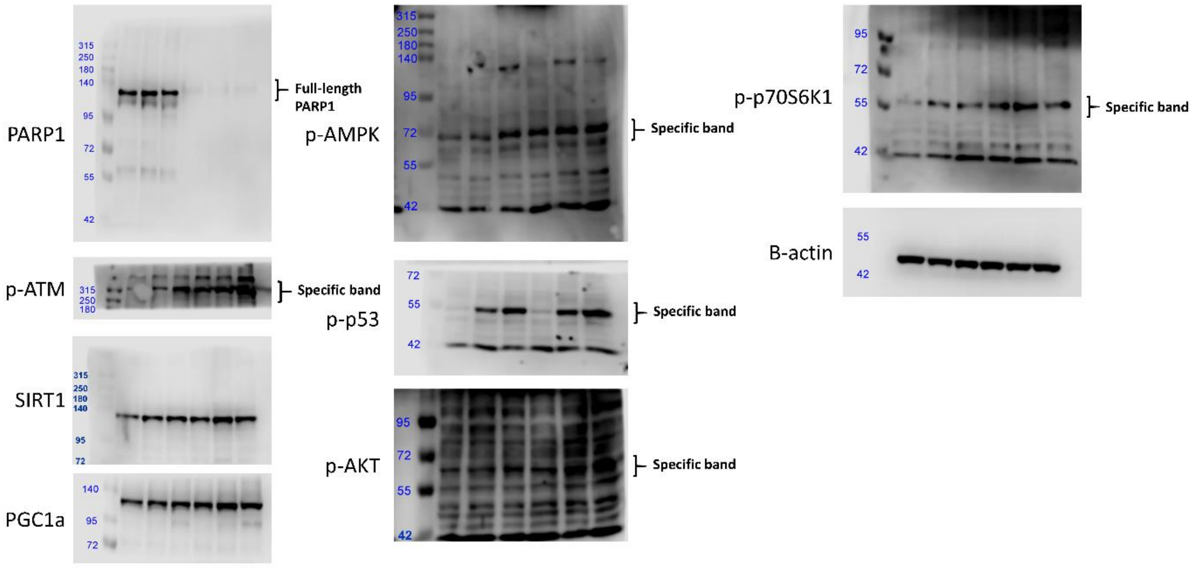

Figure 9. western blots (PARP1 and ATM inhibition)

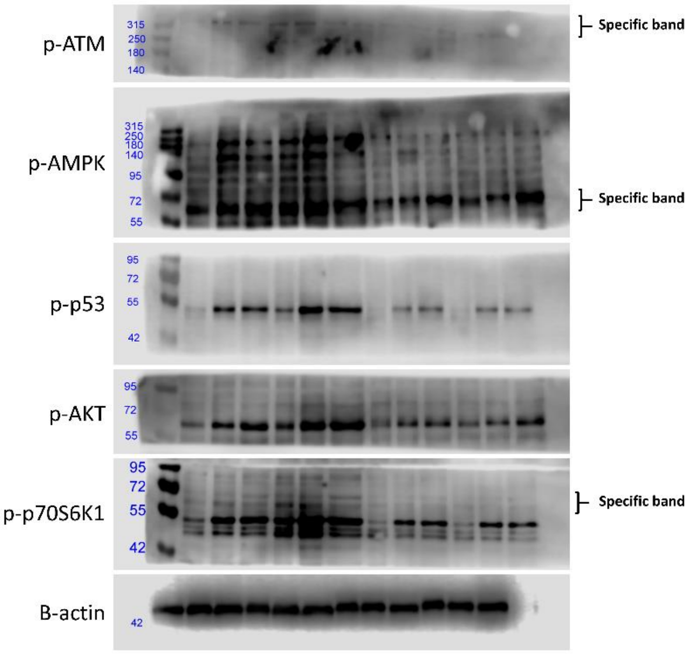

Figure 10. western blots (ATM knockdown)

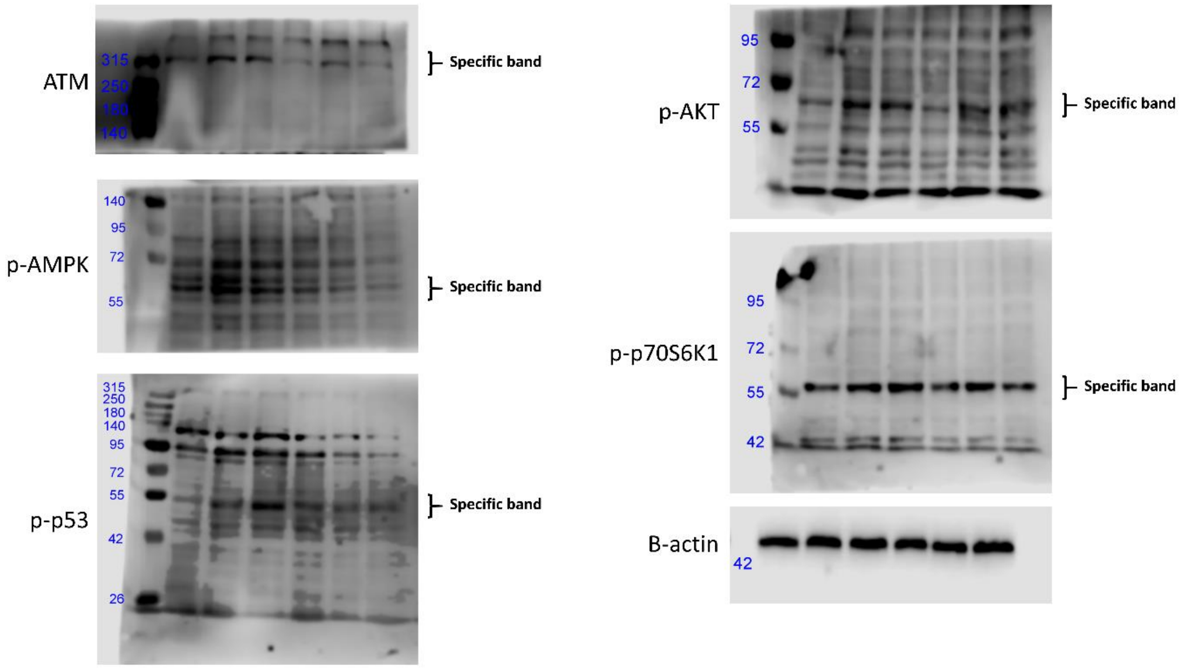

Figure S2. Sample uncut blots.
